# Effects of diffusion of innovations, spatial presence, and flow on virtual reality shopping

**DOI:** 10.3389/fpsyg.2022.941248

**Published:** 2022-08-09

**Authors:** Xiaojing Lu, Kuo-Lun Hsiao

**Affiliations:** ^1^Department of Marketing, School of Business Administration, Jimei University, Xiamen, China; ^2^Department of Information Management, National Taichung University of Science and Technology, Taichung City, Taiwan

**Keywords:** virtual reality (VR), diffusion of innovations theory (DIT), flow experience, spatial presence, purchase intention

## Abstract

Virtual reality (VR) has developed rapidly, drawing more businesses to such development. Based on the diffusion of innovations theory (DIT), the study combines the flow theory and the satisfaction perspective to explore purchase intention influencing customers’ adoption of the VR shopping platform system. This study found that satisfaction and flow experience enhance their purchase intention. In technological characteristics, relative advantage, service compatibility, spatial presence, and complexity are important in satisfaction. Among them, both relative advantage and spatial presence impact flow experience. Additionally, a cluster analysis based on gender was conducted, and the study found a significant difference between relative advantages, service compatibility, and complexity in women and men users. The flow experience is an important factor affecting women users’ shopping intention, while insignificant for male users. The implications of these findings are discussed.

## Introduction

Over these years, the surging tide of virtual reality (VR) has spread worldwide and has prompted successive investments in product development from many global enterprises (Petit et al., 2022). Companies like Google, HTC, Samsung, Sony, etc., have been vigorously devoted to education and training, shopping malls and other VR-related fields have gradually become the center of world focus (Yang and Han, 2020). Driven by the rapid trend of VR technology development, global business service-related industries have begun to transform and actively build virtual experience scenes. New operation modes, such as virtual to real, virtual incorporating with real, virtual-real value, and other new business activities, are looking forward to creating diversified business opportunities (Martínez-Navarro et al., 2019; Pizzi et al., 2019).

Various innovative shopping modes provided by combining emerging technologies (such as VR technology) brought benefits to businesses and, at the same time, improved users’ online shopping experience (Martínez-Navarro et al., 2019; Pizzi et al., 2019, 2020; Wei et al., 2019; Yang and Han, 2020). The richer media feedback resulting from the interactivity of the VR technology allows many businesses to apply the technology to various fields, such as e-commerce platforms, the retail sector, manufacturing factories, and tourism (Pizzi et al., 2020; Rahimizhian et al., 2020; Yang and Han, 2020). Pizzi et al. (2020) hold that VR technology affects people’s perceived feelings and improves their experience of the shopping situation. Pleyers and Poncin (2020) further pointed out that customer experience tends to be affected by his/her cognition, emotions, perceptions and other mental factors, promoting behavioral intention. Therefore, the industry also realizes that the shopping situation created by VR changes the usual way users interact with the platform or environment and helps to provide customers with a flow experience, which in turn affects their usage and purchase behavior (Martínez-Navarro et al., 2019; Yang and Han, 2020). The industry began to jump the wagon to join the VR business shopping environment camp.

As an immersive technology, VR provides a flow experience and simulates real situations, allowing people to immerse themselves in various sensory experiences similar to spatial presence (Su et al., 2022). As far as VR adoption is concerned, a pleasant experience is the most common state when users experience VR services (Kim and Hall, 2019; Kim and Ko, 2019). According to previous research, flow theory has been one of the most adopted approaches to exploring pleasant experiences (Kim and Ko, 2019). Based on the interpretation of the hedonic perspective derived from the flow theory, the study aims to explore its relationship to the users’ behavioral intention when making online shopping decisions through VR.

Features like immersion, interactivity, and imagination characterize VR technology, allowing users to experience spatial presence in the environment by creating a 3D 360° panoramic perspective of the merchandise and the spatial environment (Rahimizhian et al., 2020; Yang et al., 2021). Through real-time visual feedback of dynamic interaction, the spatial presence provided by VR technology produces a sense of realism in the overall virtual environment, which increases satisfaction. Eventually, it promotes purchase intention (Rodríguez-Ardura and Meseguer-Artola, 2016; Yang et al., 2021). In their exploration of virtual shopping stores, Pizzi et al. (2019) pointed out that spatial presence significantly impacts customers’ shopping experience. In light of this, we may conclude that spatial presence is one of the primary factors motivating people to adopt VR.

On the other hand, technological characteristic tends to influence people’s decision to adopt a new technology product or service. According to the diffusion innovations theory (DIT) proposed by Rogers (1995), the perceived innovative characteristic of new technology plays an influential role in adopting an emerging service or product. They also concluded that two parts constitute the innovative characteristics of technology, benefits, and cost. The findings of many scholars (Lin and Lu, 2015; Lin, 2016) on IT product or services adoption have demonstrated that the relative advantages (RAs), compatibility, and complexity will significantly affect IT adoption. The research claims that VR technology has unique positive attributes like immersion, interactivity, and imagination. In contrast, on the opposite side, the learning cost that users need to pay (cognitive effort) is the negative attribute of VR technology. Hence, by adopting the diffusion of innovations theory perspective, the research aims to understand the positive and negative service attributes that produce people’s perception of VR.

The theory of diffusion innovations has been the common approach used to explore the usage behavior of emerging technologies. As many previous VR-related theories pointed out, spatial presence and flow theory are important, influential factors (Kim and Ko, 2019). However, only a few studies discussed people’s intention to use VR technology by integrating DIT and flow theory, spatial presence, and satisfaction. The research claims that the antecedents should be included in the discussion when using new IT (such as VR) to promote customers’ purchase intention. Customers’ shopping behavior can only be enhanced by understanding whether or not customers’ intention to adopt new technology services can meet the needs of consumers. Based on DIT, and by combining spatial presence, flow theory, and satisfaction perspective, the research intends to develop and verify the factors of people’s VR shopping intention.

## Research model and hypotheses

**FIGURE 1:**
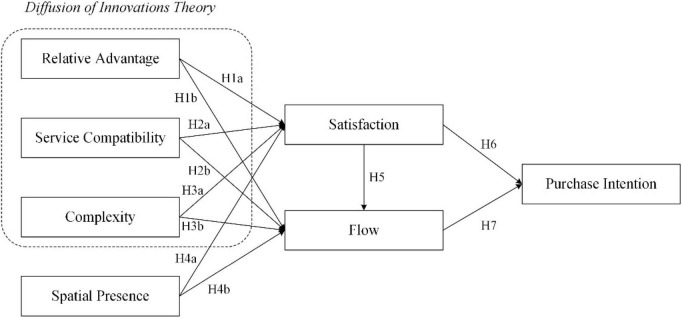
Research model.

Figure 1 is an extended research model based on the DIT by adding flow theory and satisfaction.

The DIT proposed by Rogers (1995) contends that adopting products or services tends to be affected by the cognition of the innovative characteristics. When adopting an emerging service or product, the intention of usage tends to be affected by five technological characteristics: observability, trialability, compatibility, complexity, and RA. The characteristics of DIT have been widely used in many innovative services or product issues. In the research on the influential factors impacting mobile payment, Kaur et al. (2020) demonstrated that complexity, compatibility, and RAs would positively impact usage intention and prompting usage. Ali et al. (2020) found that compatibility and complexity impact users’ intention to adopt cloud technology. While research on whether smart retail technology affects people’s perception of shopping value and the new retail technology adoption, Adapa et al. (2020) showed that RAs and complexity would affect users’ perception of value, further enhancing their behavioral intention. Previous research in IT (Lin and Lu, 2015; Lin et al., 2020) pointed out that only RAs, complexity, and compatibility would significantly influence the adoption of technology products or services. Therefore, the study adopts the RAs, compatibility and complexity mentioned in the DIT as the influential factors of technological characteristics. In addition, many scholars (Rodríguez-Ardura and Meseguer-Artola, 2016; Kim and Ko, 2019; Yang et al., 2021) pointed out that spatial presence is the most significant technological feature of VR technology. An environment composed of specific virtual objects may be created by utilizing VR technology, allowing users to view the merchandise when surrounded by a 360° panoramic situation. VR is currently an innovative style of service. Based on the above statements and by adopting the RAs, compatibility, and complexity mentioned in the DIT, and by including spatial presence as an influential factor of technological characteristics, the study intends to understand the purchase behavior intention that influences users to adopt VR in the shopping environment.

Other scholars (Kim and Hall, 2019; Kim and Ko, 2019) in VR-related research pointed out that flow experience and satisfaction are the two most important factors affecting VR technology use. In VR, users operate the preview environment to watch merchandise *via* a PC or mobile device to experience the service in a realistic scene. VR environment helps improve the user’s inner satisfaction and allows the user to immerse in the VR environment (Yang et al., 2021). In addition, other usage behaviors such as adoption and purchasing may also be produced as users have a pleasant experience (Kim and Hall, 2019; Pizzi et al., 2020). Because of this, the research is based on the DIT and develops an integrated research model framework based on the technological characteristics (RA, compatibility, complexity, and spatial presence) brought by the user’s experience of VR technology. Hopefully, we can explore user satisfaction and flow experience clearer and further drive the influential factors that enable them to purchase through VR.

### Technological characteristics

#### Perception of relative advantage

Rogers (1995) defined RA as the relatively higher benefits brought by emerging IT than the existing ones perceived by the users (Lin and Lu, 2015; Lin et al., 2020).

Relative advantage has been widely used to discuss new IT products or services, e.g., the influence of online users’ purchase intentions. Findings showed that RA directly impacts users’ attitudes (Jamshidi and Kazemi, 2019; Lin et al., 2020). Jamshidi and Kazemi (2019) research on the usage of credit cards found that RA will influence consumer satisfaction and their adoption intention. In researching the effect of eco-labeled products on people’s shopping intentions, Choshaly (2019) found that RA affects customers’ intention to purchase the goods.

On the other hand, Calvo-Porral et al. (2017) pointed out that perceptual focus on the entertainment aspect and information content has the same effect on people as the flow experience. Through VR technology (e.g., 3D modeling and 360° shopping environment), customers in the shopping situation are assisted in having an intimate feeling toward the reality which matches closer to their personal preference (Martínez-Navarro et al., 2019; Wei et al., 2019; Yang and Han, 2020). Therefore, the VR shopping environment brings the real experience of the overall virtual environment and allows the users to obtain a better shopping experience. The study holds that the RA of VR technology lies in the 360° shopping environment. In contrast to traditional e-commerce websites, it provides richer content, more visual flexibility and varied viewing angles, allowing consumers to view the merchandise through different aspects (Martínez-Navarro et al., 2019). Eventually, the RA may improve the users’ satisfaction, make them dive into the flow experience, and prompt the purchase intention. Hence, the following hypotheses were proposed:

**H1a:** The perception of relative advantage in VR shopping will positively influence users’ satisfaction.

**H1b:** The perception of relative advantage in VR shopping will positively influence users’ flow experiences.

#### Perception of service compatibility

Compatibility means that individuals use new IT services or products based on previous experience (Rogers, 1995). A new concept of service compatibility was later extended from previous research, which claimed that users might obtain the same service experience through different devices (Lin and Lu, 2015; Lin et al., 2020). Thus, the study adopts the term service compatibility in place of compatibility.

Many scholars have widely used service compatibility (e.g., Chen et al., 2018) in other IT research to investigate the acceptance intention of E-magazines. The findings showed that service compatibility has an impact on user satisfaction. Research on the factors that influence social networks shows that service compatibility directly impacts the users’ hedonic feelings (Lin and Lu, 2015). Lin (2016) further pointed out that service compatibility impacts users’ satisfaction, enhancing their behaviors.

This study holds that by integrating online or offline resources, VR technology into the shopping platform provides users with more diversified service applications (Martínez-Navarro et al., 2019). More interactive activities provided by VR shopping platforms or shopping environments allow customers to have more opportunities to access business information or services they need (Pizzi et al., 2020). Hence, the research deduces that when customers perceive the feelings of engaging in real-world situations through VR services, their satisfaction, flow experience, and purchase intention will be increased. Therefore, the following hypotheses were proposed:

**H2a:** Perception of service compatibility will positively affect users’ satisfaction.

**H2b:** Perception of service compatibility will positively affect users’ flow experiences.

#### Perception of service compatibility

Complexity perception refers to the degree to which a product/service is personally perceived to be relatively difficult to use (Lin and Lu, 2015; Jamshidi and Kazemi, 2019; Kaur et al., 2020; Rahimizhian et al., 2020).

Complexity has been widely used in discussions on emerging technology services or products. Suppose the system interface is difficult to operate for emerging products or services. It will decrease users’ satisfaction with the product and negatively impact the product or service (Lin, 2016; Jamshidi and Kazemi, 2019; Rahimizhian et al., 2020). Jamshidi and Kazemi (2019) found that the complexity of using innovative technology or service hurts user satisfaction. Kaur et al. (2020) found that complexity harms users’ mobile technology adoption, reducing the intention to recommend said mobile technology to relatives and friends. Hence, the complexity of the operating information system negatively impacts users, affecting users’ intention to adopt. Therefore, we assumed that when users conduct shopping services through VR, non-fluency, difficult-to-operate, not intuitive enough, and other factors exist in the human interface of the service system have an impact on user satisfaction with VR technology. Whereas, if the operational process causes difficulty for users to focus or get involved due to its relative difficulty in understanding, it affects users’ flow activities and behavior. Consequently, the study considers that complexity hurts VR user satisfaction and flow experience. Hence, we proposed:

**H3a:** Perception of complexity will negatively affect users’ satisfaction.

**H3b:** Perception of complexity will negatively affect users’ flow experiences.

#### Spatial presence

Spatial presence means the user’s perception of the similarity of the virtual environment to the real world (Kim and Ko, 2019; Wei et al., 2019). VR built in a 3D space provides users a 360° angle to view the merchandise and the spatial landscape. Many scholars (Rodríguez-Ardura and Meseguer-Artola, 2016; Kim and Ko, 2019; Yang et al., 2021) pointed out that spatial presence is one of the most distinctive VR technological features impacting users’ perceptual experience. Thus, the study claims that spatial presence is one of the essential technological characteristics of VR.

Cowan and Ketron (2019) found that spatial presence affects consumer satisfaction, purchase behavior, brand loyalty and corporate word-of-mouth reputation. In their research on users’ experience and behavior in VR amusement parks, Wei et al. (2019) showed that VR spatial presence positively impacts users’ satisfaction and behavioral intention. Kim and Ko (2019) discussed the VR application to sports media and found that spatial presence positively impacts flow experience. Rodríguez-Ardura and Meseguer-Artola (2016) found in their study on online learning that spatial presence would significantly influence users’ flow experience. Thus, the research contends that the characteristic of spatial presence provided by VR technology enables users to experience the service type displayed by new information, enhancing users’ satisfaction and the sense of flow. In summarizing the above viewpoints of the scholars, the study proposes the following hypotheses:

**H4a:** Spatial presence positively influences satisfaction.

**H4b:** Spatial presence positively influences flow experiences.

### Satisfaction

Satisfaction is the degree of positive or negative emotions users feel after experiencing a product or service compared to their initial expectations (Lin, 2016). When the perceived quality or performance of the product or service meets or exceeds customers’ initial expectations after usage, they will show positive emotions and satisfaction (Rahimizhian et al., 2020).

Many studies have shown that satisfaction is one of the important indicators affecting users’ attitudes and behavioral intentions (Bölen, 2020; Rahimizhian et al., 2020). Dash et al. (2021) found that satisfaction positively impacts consumers’ purchase intentions. A study by Zhu et al. (2020) on purchase intention with the stimulus-organism-response (SOR) framework found that satisfaction positively impacts consumers’ purchase intention. Many scholars (Calvo-Porral et al., 2017; Attar et al., 2020; Rahimizhian et al., 2020) concluded that IT usage often accompanies satisfaction, affecting users’ cognitive and behavioral intentions. Calvo-Porral et al. (2017) pointed out that when users are satisfied with IT services, the flow experience will enhance their behavioral intention. Findings of e-commerce-related research found that technological media tools positively impact users’ cognitive feelings and satisfaction and further promote purchase intentions (Attar et al., 2020). Therefore, the study considers that the introduction of VR into an e-commerce platform helps users obtain a sense of satisfaction, enhancing the flow experience and further driving the purchase intention behind the user’s behavior. Hence, we proposed:

**H5:** Satisfaction will influence flow experiences positively.

**H6:** Satisfaction will influence purchase intention positively.

### Flow experience

Csikszentmihalyi (1975) held that a flow experience tends to arise in people who focus intensively on a certain activity or object to the extent that other peripheral matters are neglected, further affecting their intention to use. Previous studies further pointed out that people who are intensely focused on and fully engaged in a certain activity or an object, to the extent that other unimportant perceptions are excluded, tend to absorb themselves in a state of flow (Csikszentmihalyi, 1975; Kim and Ko, 2019). When people enjoy the fun of certain activities *per se* and are even willing to devote time and energy to ignore other things, it will further affect their behavior (Kim and Hall, 2019; Kim and Ko, 2019).

Flow experience has been considered a critical factor in influencing consumers’ VR technology usage (Kim and Hall, 2019; Kim and Ko, 2019; Yang and Han, 2020). Users can watch the merchandise in a VR setting by operating the system’s preview function *via* a PC or mobile device. VR techniques allow them to immerse themselves in the simulated environment (Kim and Hall, 2019). The fun and joyful experience they gain from use will enhance their behavioral intention (Yang and Han, 2020). Hence, we proposed:

**H7:** Flow experience positively influences purchase intention.

## Research method

### Survey process

The target subjects were users who have used a VR-based shopping platform system in Taiwan. Data were collected mainly *via* an online questionnaire survey. Invitation messages were posted on popular VR forums for 8 weeks. To encourage a response rate, we offered 80 gift coupons worth US$5 each to respondents who completed the questionnaire. In order to avoid replications, respondents’ identities were checked by mailing addresses and IP addresses.

Eventually, 292 valid questionnaires were confirmed among 336 with 44 invalid ones. Among all respondents, males account for 63.7%. The major age group fell between 19 and 25 years old, accounting for 35.6%. Most have a university/college degree, accounting for 58.9%. Office workers occupied the largest group, accounting for 45.8%.

### Research instruments

In order to enhance the construct validity, construct items were mainly selected from those of the previous studies. The selected items were slightly modified to fit the VR context. The items used to measure purchase intention were adapted from Alalwan (2018). The items used to measure flow experience were modified from Chang and Zhu (2012) and Rodríguez-Ardura and Meseguer-Artola (2016). The items used to measure satisfaction were modified from Lin (2016). The one measure for spatial presence was adapted from Rodríguez-Ardura and Meseguer-Artola (2016). Items concerning RA, service compatibility, and complexity were modified from those used by Fang et al. (2017) and Kim and Ammeter (2014), and Lin and Lu (2015). Finally, all items were measured using the 5-point Likert scale, ranging from 1 (strongly disagree) to 5 (strongly agree).

## Analysis results

### Measurement model analysis

The measurement model was further assessed for construct reliability, convergent validity and discriminant validity. The reliability analysis uses composite reliability (CR) to evaluate the model’s internal consistency. The CR of each aspect of this study is above 0.8, which is higher than the recommended 0.7 by Fornell and Larcker (1981). Therefore, the measurement items of various constructs have good reliability and stability.

In order to enhance convergent validity, Bagozzi and Yi (1988) suggested three criteria: (1) all indicator factor loadings must exceed 0.5; (2) the CR should be above 0.7; and (3) the AVE of each construct should exceed 0.5. Results show that the loadings in the model exceed 0.7, the CR is 0.849–0.936, and the AVE is 0.628–0.847. Therefore, all conditions for convergent validity have been met.

Regarding discriminant validity, according to Fornell and Larcker (1981), the AVE of the construct itself is greater than the correlation coefficient of other constructs. The AVE of all constructs is greater than the square of the correlation coefficient value between constructs. Accordingly, various constructs in the measurement model are different, which also verifies the discriminant validity of various constructs. To sum up, the measurement model of the research model shows good reliability, convergent validity and discriminant validity.

### Structural model analysis

**FIGURE 2:**
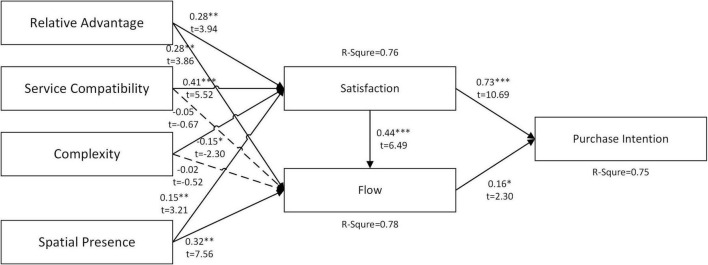
Results of the full sample. ***P < 0.001, **P < 0.01, *P < 0.05, ns – not significant.

The proposed structural model is tested and verified by SmartPLS 3.2. Figure 2 shows each path’s standardized path coefficients, path significances, and variance explained (*R*^2^). The results of path analysis show that flow experience significantly impacts all assumptions, with service compatibility and complexity (H2b, H3b) as the two exceptions.

In the aspect of variance explained (*R*^2^), purchase intention is affected by satisfaction and flow experience, with an explanatory power of 75%. Satisfaction is influenced by RAs, service compatibility, complexity, and spatial presence, with an explanatory power of 76%. Moreover, the flow experience is influenced by satisfaction, RAs, service compatibility, complexity, and spatial presence, with an explanatory power of 78%.

### Multiple-group analysis

**FIGURE 3:**
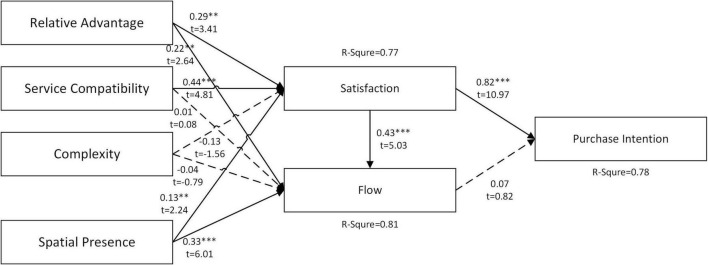
Results for men samples. ***P < 0.001, **P < 0.01, *P < 0.05, ns – not significant.

We use a multiple-group path analysis in SmartPLS software to understand the difference in the causal relationship between men (*N* = 186) and women subjects (*N* = 106). Figures 3 (male) and 4 (female) are the estimated path coefficients and variance explained (*R*^2^) of the relationship between research constructs, respectively.

The results show that gender as a subgroup has a significant difference in relative advantage → satisfaction, relative advantage → flow experience, service compatibility → satisfaction, complexity → satisfaction, flow experience → purchase intention (H1a, H1b, H2a, H3a, and H7). Regarding purchase intention, satisfaction directly impacts men subjects, while satisfaction and flow experience directly impact women subjects. Generally, we may infer that when compared with women subjects, most men are more rational than their counterparts. As shown in the findings, men tend to emphasize after-use feelings they get from operating VR technology, which then affect purchase intention through satisfaction. Whereas, for women, apart from focusing on feeling after experiencing the system, they are also affected by the entertainment aspect of the VR technology. In addition to satisfaction, online shopping intention is also influenced by their flow experience. Moreover, the findings further found that as far as emerging technologies are concerned, the operation methods, interfaces and other factors of VR technology have no direct negative impact on men users. While for women users, we found that complexity does hurt their satisfaction. Past research also indicated that male users have higher perceptions of sense of being there, spatial presence, and realness in the virtual environment (Felnhofer et al., 2012). Stanney et al. (2020) also found that female users will have more chance of having motion sickness while using VR headsets. Therefore, we conclude that male users quickly grasp new technologies than female users.

## Discussion

In the study, a research model extended from DIT, combining flow theory and satisfaction perspective, is used to verify the effects of behavior in the VR shopping environment. The findings show that RAs, complexity, compatibility, spatial presence, flow experience, and satisfaction play an important role in customers’ purchase intentions in a VR environment.

Figure 2 exhibits the results of all VR users. The results found that satisfaction and flow experience are the primary factors that prompt people’s purchase intentions through VR. Between the two, satisfaction strongly affects users’ purchase intention. The findings are similar to the research of many scholars (Attar et al., 2020; Rahimizhian et al., 2020; Zhu et al., 2020). When customers feel that the system platform meets their needs, a positive feeling will arise, affecting their behavioral intention.

Meanwhile, the result that satisfaction directly and positively influences flow experience is the same as the results in the research of Calvo-Porral et al. (2017). The research concludes that when consumers acquire positive feelings in the VR technology environment, they tend to focus better on the VR shopping environment and increase their sense of pleasure. On the other hand, the flow experience also affects users’ intention to purchase merchandise/services after VR adoption (Kim and Hall, 2019; Kim and Ko, 2019; Yang and Han, 2020). The main reason lies in the sense of exploration and fun created by the VR environment during the shopping tour, enabling consumers to immerse themselves in it, thereby enhancing their purchase intention.

Regarding technological characteristics, RA directly impacts consumer satisfaction, which is consistent with the results reached by Jamshidi and Kazemi (2019). Thus, it can be seen that consumers’ emotional feelings in using the system will be affected by the benefits gained from an emerging technology service, thereby enhancing their VR technology experience, further increasing their intention to purchase the merchandise *via* VR technology. Similarly, RA has direct positive effects on users’ flow experience. The study contends that the 3D shopping environment created by VR technology allows consumers to experience the simulated world in the spatial presence and a chance of interactivity. Eventually, it allows consumers to gain a shopping experience they have never had before.

**FIGURE 4:**
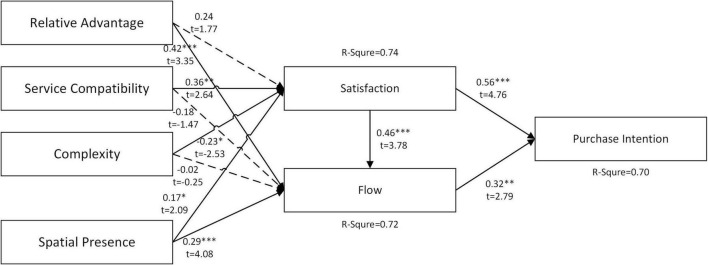
Results for women samples. ***P < 0.001, **P < 0.01, *P < 0.05, ns – not significant.

In the results of Figures 3, 4, it was found that men’s satisfaction with the VR shopping platform had a stronger influence on purchase intention, and their degree of flow on the platform had a lower influence on purchase intention. In contrast, women’s flow level on the platform strongly influences purchase intention, indicating that women will decide whether to purchase in a VR environment according to their flow experience. The complexity of the platform has less impact on men’s satisfaction but more on women’s satisfaction. Male satisfaction is more likely to be influenced by the RAs of the platform. Therefore, if the platform attracts female users, the developer should reduce the complexity of the user interface. The RA should be improved to attract more male users. The analysis results in Figures 3, 4 also show that the explanatory power of the research model for both male and female purchase intention is high (*R*^2^ > 0.7).

## Conclusion

Most of the previous literature used the DIT to explain why users adopt a certain technology service or product. However, with technology services or products as the influence factors, the DIT is short of factors for measuring consumers’ mental reactions. Therefore, this study constitutes the research’s theoretical basis to integrate both the flow theory and the satisfaction perspective. Many scholars (Rodríguez-Ardura and Meseguer-Artola, 2016; Yang et al., 2021) pointed out that spatial presence is the most prominent technological feature of VR technology. In order to extend the technological characteristics of DIT, the research includes spatial presence as an influential factor of technological characteristics.

Next, the research results found that satisfaction and flow strongly positively affect VR shopping intention, with an explanatory power of 75%. Consumers’ feelings toward operating VR technology affect their behavior, such as practicality and entertainment, which affects their decision-making of purchase intention. Accordingly, this part may provide subsequent scholars on VR technology a new angle to include satisfaction and flow experience as two additional influential factors. Hence, the research suggests that when designing VR content and system operational process, the industry needs to consider whether it meets the information content of the customer group and whether the system operational method can improve customer satisfaction.

Concerning technological characteristics of DIT, the research found that RA would positively affect satisfaction and flow experience. It can be seen that VR technology does bring different shopping experiences to the consumers, mainly because of its technological characteristics, such as multiple perspectives of previewing merchandise and real-time accessibility of product information. Thus, those new shopping ways that are different from past e-commerce websites will enhance consumers’ shopping experience. Additionally, service compatibility positively influences user satisfaction. From here, we can see that operational methods of emerging technologies must meet customers’ expectations for quickly grasping the operational process. The research reasons that the shopping situations designed by the industry on the VR technology platform should consider providing multiple service applications by integrating both virtual and real worlds. Allowing customers to engage in business with the real world through VR services increases consumers’ satisfaction with the VR shopping platform system.

On the contrary, complexity negatively impacts consumer satisfaction. Therefore, it is suggested that an emerging technology product heed the technological operation interface, system response speed, etc. When the visual feedback is too slow, it may decrease the adoption of emerging technology and affect users’ intention to use VR to shop on the platform. Thus, the industry is urged to consider the operational process, smooth service, intuitive human interface and other conditions when designing the VR technology platform to improve consumers’ satisfaction with the VR shopping platform system.

Finally, the research found that spatial presence does have a positive impact on satisfaction and flow experience. Therefore, it concluded that the multi-dimensional space of VR can improve consumers’ satisfaction with the VR shopping environment, bring a sense of spatial presence to the users, and further drive their intention to shop in the VR environment. Most of the multi-dimensional space characteristics established by VR technology allow consumers to browse the target merchandise in a 360° perspective and space, obtain information, interact with the environment, and enhance their satisfaction and flow experience.

## Data availability statement

The raw data supporting the conclusions of this article will be made available by the authors, without undue reservation.

## Author contributions

XL contributed to the research topic, methodology, experimental design, and results. K-LH contributed to the statistical analysis and discussion. Both authors contributed to the article and approved the submitted version.
